# Sensory attributes, dog preference ranking, and oxidation rate evaluation of sorghum-based baked treats supplemented with soluble animal proteins

**DOI:** 10.1093/jas/skac191

**Published:** 2022-05-23

**Authors:** Krystina A Lema Almeida, Kadri Koppel, Charles G Aldrich

**Affiliations:** Department of Grain Science and Industry, Kansas State University, Manhattan, KS 66506; Center for Sensory Analysis and Consumer Behavior, Department of Food, Nutrition, Dietetics and Health, Kansas State University, KS 66502; Department of Grain Science and Industry, Kansas State University, Manhattan, KS 66506

**Keywords:** beagle dogs, descriptive panel, hexanal, oxidation, palatability, ranking test

## Abstract

Treats are offered to dogs to reinforce the animal–owner bond and as rewards. Wheat, which contains gluten (gliadin and glutenin proteins), is often used in treats. The United States is a leading producer of sorghum which might be an alternative; however, it does not have functional properties to form viscoelastic doughs, because it is mainly composed of kafirin protein. Therefore, the objectives of this study were to determine the effects of supplementing soluble animal proteins in whole sorghum rotary-molded baked dog treats on dog preference, sensory attributes, and oxidation rate. The treats were produced in triplicate in a 2 x 4 + 1 augmented factorial arrangement of treatments. Two whole sorghum flours (WWS and WRS), four protein sources [none (NC), spray-dried plasma (SDP), egg protein (EP), and gelatin (GL)], and a positive control with wheat (WWF-GTN) were evaluated. A preference ranking test with twelve dogs was performed. Additionally, five trained panelists scored the intensity of appearance, aroma, flavor, texture/mouthfeel, and aftertaste attributes. Finally, the treats were stored at 30 °C and 60% RH, and hexanal concentrations were measured on days 0, 28, 56, and 112. The data was analyzed using the statistical software SAS for the animal and oxidation rate evaluations with significance considered at *P*<0.05. The descriptive sensory evaluation data was analyzed using multivariate analysis (XLSTAT). The dogs did not detect differences among WWF-GTN, WWS, or WRS treats when evaluated together. However, the WWF-GTN, WWS-SDP, and WWS-EP treatments were preferred among the white sorghum treatments. The EP treatments led to some consumption difficulties by dogs because of their hard texture. The panelists reported a high degree of variation in the appearance and texture across treatments. The WRS and WWS treats with SDP or EP were darker, while NC treats had more surface cracks. Initial crispness, hardness, and fracturability were higher in EP treatments compared to all other sorghum treatments. The predominant flavor and aftertaste identified were “grainy.” The hexanal values for all treats were <1.0 mg/kg except for the EP treatments that had higher values (2.0–19.3 mg/kg) across the shelf-life test. This work indicated that the replacement of WWF-GTN by WWS and WRS, along with soluble animal proteins like SDP or GL would produce comparable preference by dogs, oxidation rates, product aromatics, flavor, aftertaste attributes, and, at a lower degree, product texture.

## Teaser

This work indicated that the replacement of WWF-GTN by WWS and WRS, along with soluble animal proteins SDP or GL would produce comparable preference by dogs, oxidation rates, product aromatics, flavor, and aftertaste attributes, and, at a lower degree, product texture.

## Introduction

The development of new products involves many steps, including: identifying the product and market requirements, developing and testing the concept, defining and producing the prototypes, sourcing from suppliers, planning the manufacturing process, and the marketing design (Wang et al., 2012). As a key step for product development of pet treats, it is also important to assess the acceptance by dogs, their owners, and their stability through transport and storage, and the retention of nutritional quality and palatability. Most baked pet treats are produced with wheat which contains gluten (gliadin and glutenin prolamin functional proteins) that provide good dough structure, durability, and texture to the products. The United States is a leading producer of sorghum which might serve as a grain in baked treats (biscuits); unfortunately, it contains mostly kafirin prolamin protein, so breakage and texture are problems in its use. In a previous research, rotary-molded dog treats containing soluble animal proteins were successfully produced and determined to have comparable binding and physical attributes to those containing wheat (Lema, 2021).

Since dogs cannot provide verbal feedback, multiple indirect approaches have been evaluated to understand their preferences. For instance, food choice as preference or acceptance tests have been conducted with two foods offered simultaneously (two-bowl test) or a single food (single-bowl test) (Tobie et al., 2015). In these cases, the preferred food is determined by the total quantity eaten. Other researchers have explored operant testing methods in which the animal is required to show a response (press a lever) to access a food (Rashotte and Smith, 1984). However, in cases where more food options are intended to be compared, and there is no intention for the animals to consume excessive quantities of food, other approaches, such as a preference ranking test may be a better indicator of liking. The preference ranking test is a multiple-choice test that allows one to understand a preference based on multiple comparisons of ingredient aromatics and flavors and provides direction for individual foods when offered multiple times (Li et al., 2017). This technique of determining the preference of a product over other options is important considering that 44% of U.S. consumers purchase pet food and treats when their pet shows a positive attitude or behavior towards the flavor (Dornblaser, 2017).

Similarly, human perception is essential because the owner interacts with the food and the animal response (Francis et al., 2020). Most pet owners look for treats and snacks marketed as raw, natural, organic, U.S. sourced, with functional claims, using limited ingredients, and (or) exotic proteins, clean labels, and those that resemble human foods (Sprinkle, 2019). Moreover, the brand is also associated with quality and helps with the selection process. For instance, in a study conducted in New Zealand with 103 pet owners, 62% replied that they were loyal to a brand (Surie, 2014).

Further, pet owners also consider sensory attributes such as appearance and aroma, with color being the most influential purchasing attribute (Di Donfrancesco et al., 2014). Food preference can better be understood with a detailed breakdown of the sensory attributes identified in a product by a trained panel, even though the real perceptions of taste and flavor differ between humans and dogs (Koppel, 2014).

A product’s shelf-life is a period in which it maintains acceptable quality, specific functionality, and safety (Young, 2011). Low-moisture treats generally have a long shelf-life due to their low water activity that retards pathogenic and spoilage microorganism growth (Bramoulle et al., 2013). Nonetheless, loss of crispness and lipid oxidation can occur because of moisture adsorption and penetration of oxygen or light (Galić et al., 2009) during long-term storage. An appropriate package can control moisture adsorption; however, the oxidation process can still take place and is generally the main reason for quality decay (Manzocco et al., 2020). With lipid oxidation, secondary volatiles such as hexanal are produced which can impact food quality and negatively alter the organoleptic, nutritional, and shelf-life properties of a product (Jeleń and Wąsowicz, 2011). In dry pet food products, oxidation can mostly produce off-flavors and odors; however, it can also affect the animal well-being in a long term. For instance, Turek et al. (2003) observed that highly-oxidized diets fed to puppies reduced their serum vitamin E levels, total body fat, and impaired the rate of bone formation, which in turn affected their growth, antioxidant status, and some immune functions (Turek et al., 2003).

Our hypothesis for conducting this research was that the addition of soluble animal proteins will enhance the sensory attributes of rotary-molded dog treats with no impairment in the oxidation rates. Therefore, the objectives of this study were to determine the effects of whole wheat containing dog treats versus those produced with whole sorghum when supplemented with soluble animal proteins on their sensory attributes, dog preference ranking, and hexanal production during storage.

## Materials and Methods

The animal evaluation was conducted at Kansas State University Large Animal Research Center (LARC) under the Kansas State University Institutional Animal Care and Use Committee (IACUC) protocol #4277. In addition, the descriptive sensory evaluation was conducted at Kansas State University Center for Sensory Analysis and Consumer Behavior under the Institutional Review Board (IRB) protocol #5930.

### Experimental treatments

Rotary-molded baked dog treats were produced in triplicate at a pilot research facility (Cookie Cracker Laboratory, AIB International, Inc.; Manhattan, KS). The experimental ingredients included whole wheat flour (WWF-GTN) <180 µm (Ultragrain Hard, Ardent Mills, Denver, CO), whole white (WWS) and red sorghum (WRS) flours <150 µm (White Whole Grain and Burgundy Whole Grain, Nu Life, Scott City, KS), spray-dried plasma (SDP, Innomax Porcine Plasma, Sonac, Maquoketa, IA), egg protein (EP, OvaBind, Isonova, Spencer, IA), and gelatin (GL, Pro-Bind Plus 50, Sonac, The Netherlands). Each of the treatments also included cornmeal (Enriched Corn Meal Yellow, Sysco), salt (Iodized Salt, Morton Salt Inc., Chicago, IL), molasses (Rich Brown Hue [2:3 - #715:#677], International Molasses Corporation, Ltd., Saddle Brook, NJ), baking soda (Pure Baking Soda, Arm & Hammer, Princeton, NJ), nonfat dry milk (Nonfat Dry Milk Classic, Sysco), sodium bisulfite (Sodium Metabisulphite, LD Carlson Company, Kent, OH), inactive dry yeast (Nutritional Yeast, Bob’s Red Mill Natural Foods, Milwaukie, OR), and all-purpose shortening (Premium All-Purpose Shortening, Ventura Foods, Brea, CA). A negative control (NC) with no protein added was also tested (Table 1). The dry ingredients were mixed in a planetary mixer (Hobart Legacy HL800 Mixer) for 1 min at 55 rpm, then the wet ingredients were added and mixed for 2 min at 55 rpm plus 4.5–6 min at 96 rpm. The dough was molded into bone-shaped treats in a rotary molder (70 PSI Weidenmiller) and baked for 20–25 min at 375°F (Lema, 2021).

**Table 1.** Ingredient composition of the experimental treats produced by rotary molding: Positive control with wheat, whole white sorghum, whole red sorghum, negative control with no protein added, spray-dried plasma, egg protein, and gelatin.

Ingredients, %	Treatments								
	WWF GTN	WWS NC	WWS SDP	WWS EP	WWS GL	WRS NC	WRS SDP	WRS EP	WRS GL
Whole wheat flour	70.1	-	-	-	-	-	-	-	-
Whole red sorghum flour	-	-	-	-	-	68.6	69.0	65.3	69.8
Whole white sorghum flour	0	68.6	68.9	65.3	69.8	-	-	-	-
Cornmeal	17.5	19.1	12.5	11.8	12.5	19.1	12.5	11.8	12.5
Spray-dried plasma	-	-	6.22	-	-	-	6.23	-	-
Egg protein	-	-	-	11.28	-	-	-	11.28	-
Gelatin	-	-	-	-	5.35	-	-	-	5.35
Water_ (% added on top of ingredients)_	24.5	41.1	28.9	24.6	31.0	41.1	29.2	27.5	32.8

Other ingredients: molasses 5.6%, all-purpose shortening 3.5%, nonfat dry milk 2.2%, salt 0.7%, baking soda 0.4%, sodium bisulfite 0.003%, inactive dry yeast 0.003%

### Animal evaluation

The order of treat preference was evaluated according to the preference ranking test for dogs developed by Li et al. (2017). The test consisted of five different phases each of 5-d length. An acclimation phase included a null test in which commercial dog treats (Milk-Bone Flavor Snack Dog Biscuits, Big Heart Pet Brands Inc., San Francisco, CA) were provided. This was followed by two evaluations, one each for white sorghum treatments and red sorghum treatments (both compared to WWF-GTN), and a final ranking test comparing WWF-GTN to selected white and red sorghum treatments. The treatments for the last phase were chosen based on the results obtained in the two previous phases. The white sorghum treatments were reevaluated before the last phase due to a lack of dog responses on the first trial.

For this study, 12 healthy Beagle dogs (four females and eight males) aged 5.58 ± 0.23-yr old were used. They were housed under ambient environmental conditions (20 °C; 60% relative humidity) in pairs inside pens (7.8 square meter inside run with an attached 18 square meter outdoor run) on a 12-h light cycle and had access to water ad libitum. They received two main feedings per day at 0800 and 1100 h before starting the trial at 1600 h each day. The allowance of food with a short lapse between each feeding allowed to provide the animals their daily energy requirement but also avoid the animals to be full by the time of the test, which increased their interest for the ranking test. Treats from all production replicates were blended into their respective composite samples. In each test, 3.0–5.0 g of treat was placed into a numbered hollow rubber toy (Kong). Each dog was first allowed to sniff each toy + treat individually, then five toys + treats, in a randomized order, were evenly distributed on the floor in a corner of the experimental pen. The pen had an area of approximately 1.5m x 1.5m in a room that was separate from all other dogs. The room was a noise-free and smell-free environment, which eliminated the distraction from the barking and smell of the other dogs. The time (mm:ss:0) was recorded from the moment the dog was released until it ate each treat. Each empty rubber toy was picked up from the floor and its number (sample identification) was recorded. Each dog was allowed to continue with the test until all treats had been removed from the toys.

#### Statistical analysis

The ranking scores were analyzed with ANOVA Cochran–Mantel–Haenszel statistic, which is a generalization of Friedman’s test using the FREQ Procedure by statistical analysis software (SAS 9.4 Inst. Inc., Cary, NC). Then, the rank means were separated using Tukey’s HSD (Honest Significance Difference) test and considered significant at a probability of *P*<0.05 using the GLIMMIX procedure by statistical analysis software (SAS 9.4 Inst. Inc., Cary, NC).

### 2.3 Descriptive sensory evaluation

Five highly trained panelists scored the intensity of appearance, aroma, flavor, texture/mouthfeel, and aftertaste attributes of the treats. A consensus method and intensity scores were used based on a scale from 0 = none to 15 = extremely high with 0.5 increments according to the work of Di Donfrancesco et al. (2012). Each of the sensory panelists had more than 120 h of descriptive analysis panel training with a variety of products, including dry cat- and dog-food. They were trained on techniques and practices for attribute identification, terminology development, and intensity scoring.

Each sample was randomly assigned a three-digit code. For appearance, flavor, texture/mouthfeel, and aftertaste evaluation, one small treat was served in a 100-mL cup and provided individually to each panelist. For the aroma evaluation, one large treat was crushed and served (approximately 15 g) in a medium glass snifter; two panelists shared a snifter. Hot towels, cucumbers, and water were provided to assist panelists as cleanout. The evaluation was divided into three phases. On orientation day 1, the panelists smelled and tasted the samples to generate a lexicon of attributes according to Di Donfrancesco et al. (2012). Then, the panelists evaluated three treatments per day for a duration of 3 d. Finally, a single day side-by-side evaluation was conducted to confirm scores.

The attributes identified by the trained panelists were brown, color uniformity, surface roughness, and surface cracks for the appearance. For the aroma, attributes such as overall intensity, grain, musty/dusty, toasted, cardboard, stale, and sweet aromatics were detected. The identified flavors were grain, cardboard, leavening, starchy, toasted, and sweet aromatics. Moreover, the texture/mouthfeel attributes detected were initial crispiness, hardness, fracturability, gritty, cohesiveness of mass, and particles. Finally, grain, cardboard, starchy, and toasted were perceived as aftertaste attributes. All attributes were defined and anchored to the scale with reference materials as described in Di Donfrancesco et al. (2012). Surface cracks, leavening, and overall intensity were new attributes detected in this study. Surface cracks refers to the perceived amount of cracks on the surface. The reference used was a package picture of Nabisco ginger snaps cookies (7.5). Leavening refers to the flat metallic somewhat sour/bitter aromatics associated with baking soda and/or baking powder in baked flour products. The reference used was Jiffy corn bread mix (4.0). Overall intensity refers to the total intensity of all types of notes perceived. The references used were cereal mix ‘dry’ (5.0) and Lorna Doone Cookie (6.0).

#### Statistical analysis

A multivariate analysis approach was applied to the perceived attributes using XLSTAT (Addinsoft, New York, USA) and a Principal Component Analysis (PCA) was performed to differentiate the treats relative to the sensorial characteristics. To determine linear correlations across the attributes perceived, Pearson correlation coefficients were used with significance considered at *P*<0.05. Radar charts were also plotted in Excel to visualize the relationships among treatments and attributes.

### Oxidation rate evaluation

Samples were kept frozen (-18 °C) prior to this evaluation. Approximately 50 g of treats per replicate were placed into a whirl-pak bag, each with four pinholes and kept in an environmental chamber at 30 °C and 60% relative humidity for evaluation at 0, 28, 56, and 112 d. At each time point samples were removed and frozen (-18 °C) before analyzing aromatic compounds. For the sample preparation, treats were ground in a coffee grinder and 0.5 ± 0.02 g of the pulverized sample was weighed into a 10 mL screw-cap vial to which 0.99 mL of distilled water was added. The extraction of the volatiles was performed according to Koppel et al. (2013). The isolation, tentative identification, and semiquantification of the volatile compounds were performed on a gas chromatograph (GC-2010 Plus; Shimadzu, Tokyo, Japan) coupled with a mass spectrometer (MS) detector (GCMS-QP2020; Shimadzu, Tokyo, Japan). The GC-MS system was equipped with an SH-Rxi-5Sil MS cross bond column (Shimadzu, Tokyo, Japan; 30 m × 0.25 mm × 0.25 μm film thickness). The column was heated from 40 to 240 °C. The ion source was set at 200 °C and the mass spectrometer scanned for masses between 35 and 350 m/z. Volatile compounds were identified using the NIST library. All treatments were analyzed in triplicate. Hexanal was reported and calculated against 10 µL 100 ppm 1,3-dichlorobenzene as the internal standard.

#### Statistical analysis

The data processing, analysis of variance, and least-squares means separation for repeated measures across time were performed using the GLM procedure of the statistical analysis software (SAS 9.4 Inst. Inc., Cary, NC). For the least-squares means separation, Tukey’s HSD (Honest Significance Difference) test was applied and were considered significant when the probability was *P*<0.05. Two different models were generated: a one-way ANOVA comparing the nine treatments across a day and a one-way ANOVA comparing time within a treatment.

## Results

### Animal evaluation

The ranking results correspond to 10 dogs because two did not complete the study. The dog cohort size was a limitation in our study. Therefore, the results presented could be perceived different if repeated with a larger sample size. Lower values indicate more preferred treatments. In the white sorghum evaluation, the WWF-GTN, SDP, and EP treatments were comparable and preferred (*P*<0.05) over NC. The GL was less preferred than EP but equally accepted relative to the SDP and WWF-GTN treatments. In the red sorghum evaluation, there were no differences among treatments (*P*>0.05); however, lower numerical values were associated with SDP, EP, and WWF-GTN treatments. Based on the results of the individual phases, an analysis comparing the proteins SDP and GL from white and red sorghum vs. the positive control (WWF-GTN) was merited. These treatments were selected based on their similar protein content and considering the difficulties observed for the dogs in eating the EP treatments due to their harder texture. In this last comparison, no differences were found between treatments (*P*>0.05); nonetheless, lower numerical values were observed for the sorghum treatments. Also, the white sorghum treatments had lower rank values within the same protein source (Table 2). The average time the dogs took to complete the white sorghum phase was slightly shorter than the red sorghum phase (2.2%). However, unlike to what occurred in the individual phases, the average time in the combined evaluation was shorter for the WRS when compared to the WWS treatments. When average times were compared overall, they significantly decreased in the final evaluation, most likely because the dogs were more acclimated to the study procedures (Table 2).

**Table 2.** Rank order preference means, and average time of ranking phase completion of baked dog treats produced with different cereals and soluble animal proteins combinations.

Treatments	Phases					
	WWF-GTN/ WWS		WWF-GTN/ WRS		WWF-GTN/WWS/WRS	
	Rank mean	Avg. Time, mm:ss:0	Rank mean	Avg. time, mm:ss:0	Rank mean	Avg. time, mm:ss:0
WWF-GTN	2.90 ^bc^	00:23.4	2.84	00:27.6	3.35	00:13.0
WWS-NC	3.70 ^a^	00:24.6	-	-	-	-
WWS-SDP	2.84 ^bc^	00:23.6	-	-	2.75	00:14.7
WWS-EP	2.36 ^c^	00:22.7	-	-	-	-
WWS-GL	3.20 ^ab^	00:21.8	-	-	3.00	00:14.2
WRS-NC	-	-	3.28	00:32.2	-	-
WRS-SDP	-	-	2.82	00:24.3	2.78	00:12.5
WRS-EP	-	-	2.84	00:30.5	-	-
WRS-GL	-	-	3.22	00:34.0	3.13	00:13.5
Avg phase time		00:23.2		00:29.7		00:13.6
SEM	0.192		0.200		0.190	
*P*-value	0.0001		0.2822		0.1619	

a-c: Means with different lowercase superscripts within a column represent statistical difference among treatments (P<0.05)

Results correspond to 10 dogs because two did not complete the study

Lower rank means indicate more preferred treatments

### Descriptive sensory evaluation

**Figure 1.**
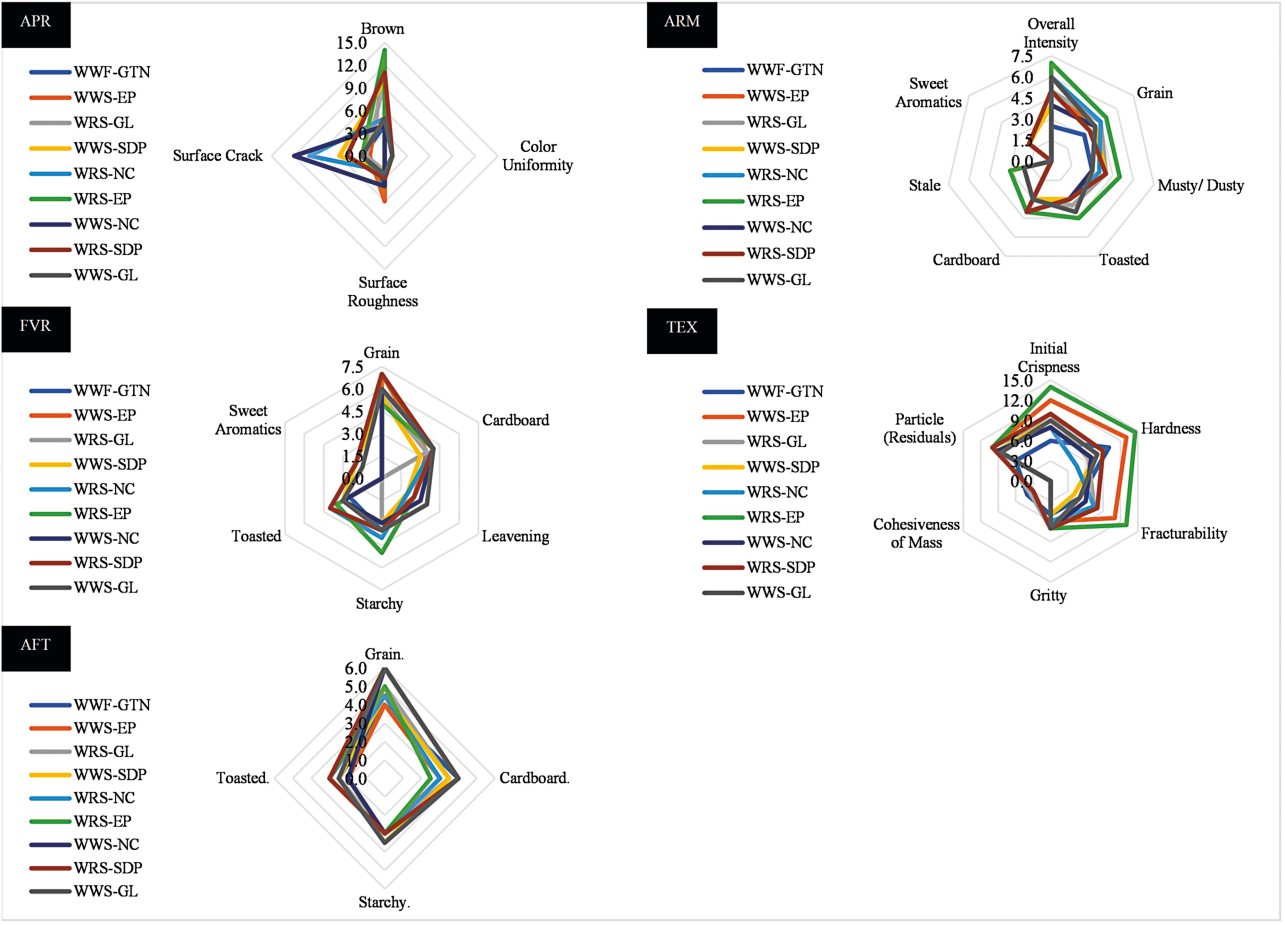
Radar chart for appearance, aroma, flavor, texture, and aftertaste attributes of baked dog treats produced with different cereals and soluble animal proteins combinations.

Brown color and surface cracks were the most differentiating appearance attributes, wherein WRS and WWS treats with SDP or EP resulted in a darker appearance (10.0–14.0), while NC treats had more surface cracks (10.0–12.0) (Figure 1). Aroma attributes did not vary substantially among samples except for the overall intensity that was higher for WRS-EP (7.0). Sweet aromatics were mostly imperceptible (< 2.0) for all treatments (Figure 1). Grainy was the most perceived flavor with values ranging from 5.0–7.0. Other flavors such as cardboard, leavening, starchy, and toasted were perceived at lower proportions (2.0–4.0), while sweet aromatics were almost unnoticed (<1.0) (Figure 1). Initial crispness, hardness, and fracturability were pronounced in EP treatments (11.0–14.5) in comparison to all other sorghum treatments (4.0–9.0). The WWF-GTN treatment was higher than SDP, GL, and NC treatments regarding hardness (10.0) but had lower initial crispiness (6.0) and fracturability (5.0). All treats had less cohesiveness of mass and more particle residuals than the control WWF-GTN (Figure 1). The predominant aftertaste of all the samples was grainy with values that ranged from 4.0–6.0 (Figure 1). All values presented correspond to intensity attributes, wherein higher values indicate a more perceived note. Refer to Section 2.3 to find the reference used for each attribute and the value given for comparison.

Based on the multivariate analysis, it was found that brown appearance had a strong positive correlation with musty/dusty aroma (r = 0.944) and initial crispiness (r = 0.891). Moreover, aroma attributes such as grain had a strong positive correlation with the overall aroma intensity (r = 0.808) and toasted aroma with stale aroma (r = 0.922). Regarding the texture attributes, initial crispiness had a strong positive correlation with musty/dusty aroma (r = 0.868) and treat fracturability (r = 0.860) (Tables 3, 4, and 5).

**Table 3.** Pearson correlation coefficients for appearance and aroma attributes from baked dog treats scored by the sensory descriptive panel.

Variables		Appearance			Aroma						
		Brown	Surface Roughness	Surface Crack	Overall Intensity	Grain	Musty/ Dusty	Toasted	Cardboard	Stale	Sweet Aromatics
**APR**	Brown	**1**	0.098	-0.515	0.443	0.437	**0.944**	0.467	0.041	0.298	0.391
	Surface Roughness	0.098	**1**	-0.179	-0.278	-0.337	-0.083	-0.014	-0.120	-0.153	-0.472
	Surface Crack	-0.515	-0.179	**1**	-0.103	0.070	-0.372	-0.534	0.620	-0.335	-0.140
**ARM**	Overall Intensity	0.443	-0.278	-0.103	**1**	**0.808**	0.543	**0.720**	0.395	**0.687**	-0.156
	Grain	0.437	-0.337	0.070	**0.808**	**1**	0.607	0.610	0.316	0.516	-0.071
	Musty/ Dusty	**0.944**	-0.083	-0.372	0.543	0.607	**1**	0.505	0.217	0.396	0.313
	Toasted	0.467	-0.014	-0.534	**0.720**	0.610	0.505	**1**	-0.024	**0.922**	-0.305
	Cardboard	0.041	-0.120	0.620	0.395	0.316	0.217	-0.024	**1**	0.163	-0.158
	Stale	0.298	-0.153	-0.335	**0.687**	0.516	0.396	**0.922**	0.163	**1**	-0.369
	Sweet Aromatics	0.391	-0.472	-0.140	-0.156	-0.071	0.313	-0.305	-0.158	-0.369	**1**
**FVR**	Starchy	0.271	-0.214	-0.093	0.638	0.486	0.496	0.605	0.538	**0.752**	-0.378
	Toasted	0.278	-0.342	0.322	0.622	0.376	0.390	-0.013	**0.754**	0.085	0.164
	Sweet Aromatics	0.011	**-0.777**	0.083	0.188	0.100	0.026	-0.270	-0.052	-0.248	**0.699**
**TEX**	Initial Crispness	**0.891**	0.167	-0.422	**0.709**	0.658	**0.868**	**0.735**	0.179	0.575	0.035
	Fracturability	**0.718**	0.363	-0.301	0.626	0.565	**0.753**	0.596	0.379	0.441	-0.240
	Particle (Residuals)	**0.702**	0.025	0.059	**0.681**	0.612	0.662	0.314	0.426	0.204	0.236
**AFT**	Cardboard	-0.611	-0.042	0.157	-0.629	**-0.767**	**-0.755**	-0.511	-0.212	-0.396	0.224

Pearson (r-values) in bold are different from 0 (*P*<0.05). Appearance (APR), aroma (ARM), flavor (FVR), texture (TEX), aftertaste (AFT)

**Table 4.** Pearson correlation coefficients for flavor attributes from baked dog treats scored by the sensory descriptive panel.

Variables		Flavor					
		Grain	Cardboard	Leavening	Starchy	Toasted	Sweet Aromatics
**APR**	Surface Roughness	0.361	0.087	0.332	-0.214	-0.342	**-0.777**
**ARM**	Cardboard	0.178	0.580	0.271	0.538	**0.754**	-0.052
	Stale	-0.531	0.338	0.386	**0.752**	0.085	-0.248
	Sweet Aromatics	-0.113	-0.574	-0.557	-0.378	0.164	**0.699**
**FVR**	Grain	**1**	0.219	0.222	-0.255	0.425	0.157
	Cardboard	0.219	**1**	0.423	0.520	0.302	-0.302
	Leavening	0.222	0.423	**1**	0.194	0.078	-0.363
	Starchy	-0.255	0.520	0.194	**1**	0.466	-0.186
	Toasted	0.425	0.302	0.078	0.466	**1**	0.459
	Sweet Aromatics	0.157	-0.302	-0.363	-0.186	0.459	**1**
**AFT**	Toasted	-0.024	0.318	-0.119	**0.725**	**0.719**	0.464

Pearson (r-values) in bold are different from 0 (*P* < 0.05). Appearance (APR), aroma (ARM), flavor (FVR), aftertaste (AFT)

**Table 5.** Pearson´s correlation values for texture and aftertaste attributes from baked dog treats scored by the descriptive panel.

Variables		Texture						Aftertaste			
		Initial Crispness	Hardness	Fracturab.	Gritty	Cohesiv. of Mass	Particle (Residuals)	Grain	Cardboard	Starchy	Toasted
**APR**	Brown	**0.891**	0.646	**0.718**	0.188	-0.064	**0.702**	-0.137	-0.611	-0.123	0.236
**APR**	Overall Intensity	**0.709**	0.177	0.626	0.613	-0.499	**0.681**	0.283	-0.629	0.024	0.486
	Grain	0.658	0.070	0.565	0.454	-0.525	0.612	0.065	**-0.767**	-0.054	0.259
	Musty/ Dusty	**0.868**	0.571	**0.753**	0.180	-0.115	0.662	-0.170	**-0.755**	-0.189	0.426
	Toasted	**0.735**	0.594	0.596	0.484	-0.302	0.314	0.138	-0.511	0.115	0.179
**FVR**	Starchy	0.442	0.404	0.576	0.218	-0.187	0.153	0.000	-0.592	-0.214	**0.725**
	Toasted	0.303	-0.167	0.385	0.380	-0.186	0.621	0.297	-0.331	-0.249	**0.719**
**TEX**	Initial Crispness	**1**	**0.687**	**0.860**	0.450	-0.341	**0.786**	0.032	**-0.713**	-0.134	0.166
	Hardness	**0.687**	**1**	**0.680**	0.008	-0.093	0.223	-0.251	-0.443	-0.170	-0.048
	Fracturability	**0.860**	**0.680**	**1**	0.399	-0.190	0.624	-0.140	**-0.776**	-0.565	0.220
	Gritty	0.450	0.008	0.399	**1**	-0.082	0.545	**0.783**	0.000	-0.218	0.123
	Cohesiv. of Mass	-0.341	-0.093	-0.190	-0.082	**1**	-0.481	-0.085	0.443	-0.374	0.166
	Particle (Residuals)	**0.786**	0.223	0.624	0.545	-0.481	**1**	0.305	-0.497	-0.089	0.137
**AFT**	Grain	0.032	-0.251	-0.140	**0.783**	-0.085	0.305	**1**	0.472	0.171	0.096
	Cardboard	**-0.713**	-0.443	**-0.776**	0.000	0.443	-0.497	0.472	**1**	0.254	-0.286
	Starchy	-0.134	-0.170	-0.565	-0.218	-0.374	-0.089	0.171	0.254	**1**	-0.081
	Toasted	0.166	-0.048	0.220	0.123	0.166	0.137	0.096	-0.286	-0.081	**1**

Pearson (r-values) in bold are different from 0 (*P*<0.05). Appearance (APR), aroma (ARM), flavor (FVR), texture (TEX), aftertaste (AFT)

**Figure 2.**
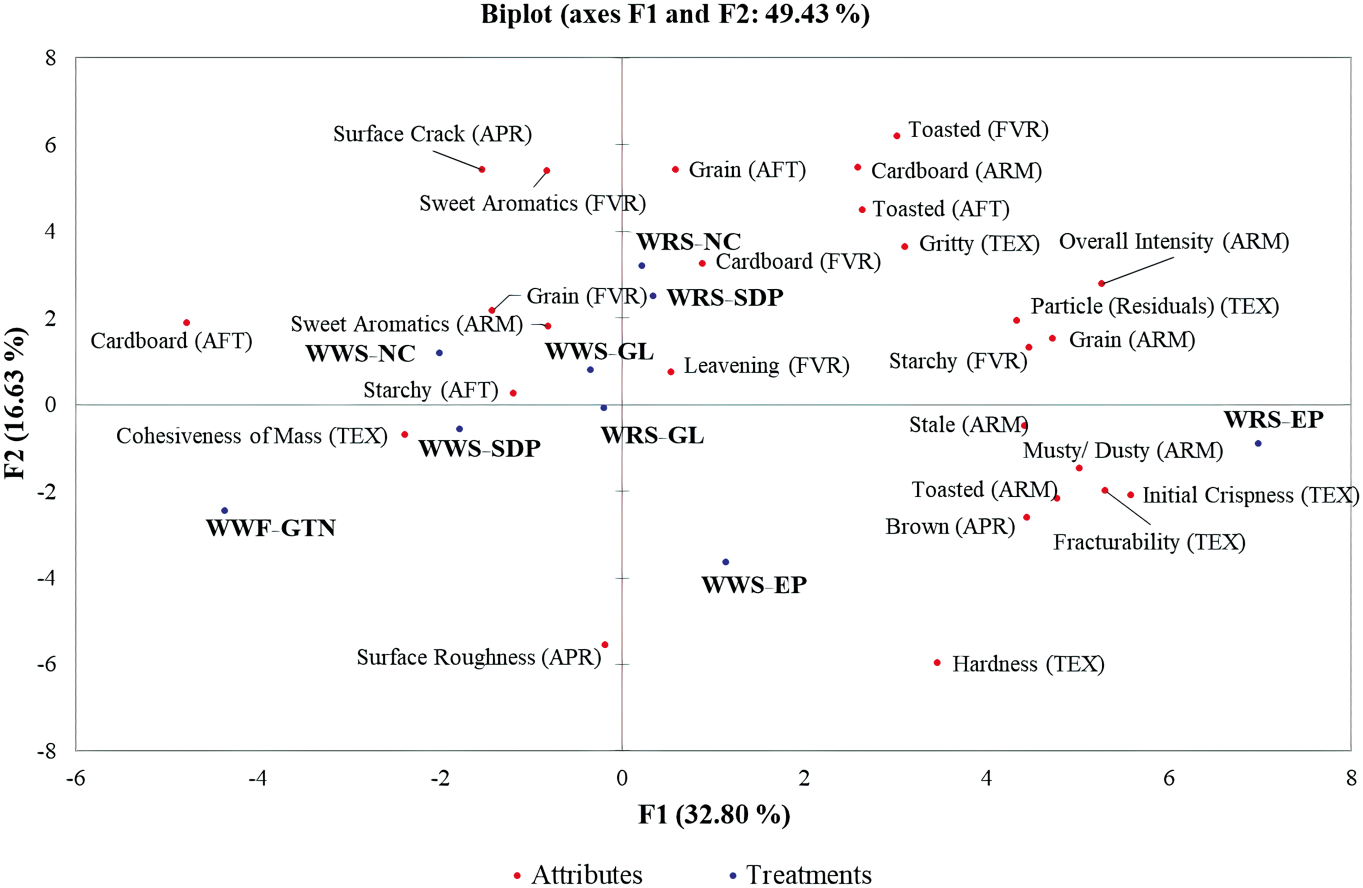
Principal component analysis of appearance, aroma, flavor, texture, and aftertaste attributes of baked dog treats produced with different cereals and soluble animal proteins combinations.

An overall picture of the attributes perceived per treatment is presented in the biplot obtained by PCA. The components F1 and F2 explained 49.43% of the variation in the dataset wherein, hardness, toasted flavor, cardboard aroma, initial crispiness, and overall intensity aroma were the attributes that explained a large proportion of the total variation or were the most differential notes across samples. The PCA clustered similarly perceived samples (NC, SDP, and GL) regarding their sensorial attributes with most of them in the negative quadrant of component 1 (F1). Nonetheless, the EP treatments were separated and located in the positive quadrant of component 1 because these treatments had higher initial crispness, hardness, and fracturability (F1). The WWF-GTN treatment was not part of the main cluster because it had higher cohesiveness of mass and hardness than the main cluster; however, it was also located in the negative quadrant of component 1 because most attributes were similar (Figure 2).

### Oxidation rate evaluation

Hexanal is an aldehyde that originates from the oxidation of unsaturated fatty acids such as linoleic acid within a food matrix. Therefore, it can be used as a marker of oxidative rancidity. The values of hexanal obtained for the treats were relatively low in all treatments (<1.0 mg/kg) except for the EP that had considerably higher hexanal concentrations (2.0–19.3 mg/kg) across the duration of the evaluation (112 d). The hexanal concentration for the EP treatments, especially when WRS was the cereal source produced a hexanal peak that was more noticeable on day 0. Contrary to what was expected, the hexanal concentrations declined over time for the WRS-EP and WRS-GL treatments. For the rest of the treatments, the hexanal values remained relatively constant throughout the evaluation timeline (Table 6).

**Table 6.** Hexanal detection (mg/kg) in baked dog treats produced with different cereals and soluble animal proteins combinations.

Treatment	Evaluation Period				SEM*	*P*-value*
	Day 0	Day 28	Day 56	Day 112		
WWF-GTN	0.18 ^b^	0.27 ^b^	0.18 ^b^	0.19 ^c^	0.036	0.2996
WWS-NC	0.98 ^b^	0.44 ^b^	0.22 ^b^	0.11 ^c^	0.430	0.5208
WWS-SDP	0.74 ^b^	0.45 ^b^	0.33 ^b^	0.31 ^bc^	0.113	0.0842
WWS-EP	7.01 ^b^	6.30 ^ab^	3.29 ^a^	2.05 ^ab^	1.806	0.2385
WWS-GL	0.57 ^b^	0.36 ^b^	0.21 ^b^	0.21 ^bc^	0.155	0.3626
WRS-NC	0.82 ^b^	0.28 ^b^	0.15 ^b^	0.20 ^bc^	0.172	0.0836
WRS-SDP	0.70 ^b^	0.49 ^b^	0.40 ^b^	0.37 ^bc^	0.080	0.0729
WRS-EP	19.37 ^aA^	9.56 ^aAB^	4.38 ^aC^	3.52 ^aC^	2.473	0.0068
WRS-GL	1.35 ^bA^	0.24 ^bB^	0.21 ^bB^	0.24 ^bcB^	0.243	0.0256
SEM**	1.477	1.312	0.516	0.375		
*P*-value**	<.0001	0.0004	<.0001	<.0001		

a-c: Means with different lowercase superscripts within a column represent statistical difference among treatments within each day (*P* < 0.05)

A–C: Means with different uppercase superscripts within a row represent statistical difference among days within each treatment (*P* < 0.05).

*: reference to treatments **: reference to days

## Discussion

### Animal evaluation

Throughout years of evolution, dogs have retained many ancestral eating behaviors. For instance, dogs rely heavily on olfactory senses when offered any food (Bradshaw, 2006; Pétel et al., 2018). Some research shows that olfactory sense is critical to discerning preferred versus non-preferred foods (Houpt et al., 1982). However, it is not well-understood whether the odors of the preferred foods are more hedonically appealing (Hall et al., 2017). Also, dogs usually do not invest much time masticating and savoring, instead they eat in a gluttonous manner (Aldrich and Koppel, 2015). Dogs possess only a fraction of the taste buds in comparison to humans (Koppel, 2014). Nonetheless, dogs can detect sour, bitter, salty, sweet, and umami flavors when stimulation of these chemoreceptors occurs. Therefore, it can be inferred that their highly developed sense of smell (>220 million olfactory receptors) contributes to a greater degree their overall flavor perception as the nose concentrates, moisturizes, and directs odorized air towards their olfactory epithelium which assures that warmed molecules are more easily detected (Castillo, 2014). In addition, dogs have different bite forces that increase with higher body weight and size of the skull which can also be influenced by the dog’s chewing enthusiasm, personality, breed, and training (Kim et al., 2018).

Dogs choose short-term food based on its palatability (Hall et al., 2018). This is influenced by a combination of taste, aroma, texture, size, appearance, temperature, and consistency (Griffin and Beidler, 1984). Moreover, their food preferences can also be determined by their genetics and early-life experiences (Bhadra and Bhadra, 2014). Our results could be explained by the combination of these factors, which were perceived by the animal after the various treatments were offered repeatedly. For instance, the EP treatments were numerically preferred over the other treatments, most likely because of a stronger aroma, especially when these treats were offered for the first time. However, these treats, particularly when combined with WRS, were quite hard and difficult to chew and consume which may have overridden the animals’ interest. Therefore, dogs may have selected different treats than one might predict as the odor alone may not have been sufficient motivation to maintain a strong response across the multiple trials. Instead, it was more important at the beginning for locating and identifying the treats rather than for consumption (Houpt and Smith, 1981), and the texture may have played an essential role regarding enjoyment while eating.

Higher moisture (lower dry matter) and lower crude fiber in dry foods are thought to boost palatability and a dogs’ food preference (Araujo and Milgram, 2004; Alegría-Morán et al., 2019). Pétel et al. (2018) demonstrated that higher moisture can increase the elasticity and the porosity of kibbles, which may contribute to greater volatile (aroma) release. In addition, higher moisture also reduces the texture, which is preferred by dogs as denoted by Kitchell (1972) when they compared canned and semimoist food to dry food, most likely due to a more pleasant mouthfeel. In our study, treat moisture did not differ across treatments with average values fluctuating between 3%–8%, this was consistent with that recommended by Bramoulle (2013).

An important observation in this study was that the addition of protein sources increased the acceptance of the sorghum treats. In both individual phases, the treatments with no added soluble animal proteins had the highest numerical values (least preferred). For this reason, the NC treatments were not included in the final comparison. According to Nagodawithana et al. (2008), the hydrolysis of proteins can help enhance a product’s acceptability. One reason could be that the biogenic and volatile amines can influence the aroma of a product. In turn, this may increase product palatability given that the aroma of a food presented before eating can increase the appetite (Zoon et al., 2016). Moreover, dogs tend to be highly sensitive to the tastes of amino acids, organic acids, and nucleotides that are mainly found in animal tissues (Case et al., 2011; Hidalgo and Takatsu, 2012).

In the preliminary phases of preference ranking tests, dogs ate the WWS faster relative to WRS and this was thought to be associated with the astringent flavor that has been reported for sorghum, especially when the pericarp is darker (House et al., 1995). Awika and Rooney (2004) indicated that red sorghums have significantly higher levels of extractable phenols than white sorghums. Nonetheless, a slightly different pattern was observed in the combined phase in which both WWS and WRS were analyzed. Thus, further investigation regarding this single parameter and with a larger dog cohort should be conducted to better understand the change.

Comparable to our study, Thompson et al. (2016) evaluated preference of dog foods in two phases. In the first phase, the dogs sniffed and observed two products without being able to eat them, while in the second phase, the dogs were allowed to consume the products. The authors observed that the proportion of time spent by the dogs exploring the foods was correlated to their consumption. In our preference ranking test, the time allowed for sniffing each toy + treat before starting the trial was not recorded; however, the dog handler displayed each of the five treatments to the dogs for approximately the same amount of time. In both instances, there was a substantial impact from aroma which supersede visual cues on dogs’ selection.

### 4.2 Descriptive sensory evaluation

The human sensory panel complemented the ranking test results and the physical measurements obtained by the instrumental analysis. Sorghum products have previously been evaluated for their sensory attributes. For example, Chiremba et al. (2009) found comparable acceptance of red tannin-free sorghum biscuits in comparison to wheat regarding liking but not texture. In our case, the panelists found similarities regarding flavor and aftertaste as “grainy” was predominant, and “overall intensity” the stronger aroma regardless of the cereal or protein used.

The panelists identified darker hues in SDP and EP treats, and also for GL when combined with WRS. Visually, the NC treatments, because of their lack of added protein had more surface fissures/cracks. Thus, the inclusion of proteinaceous ingredients verified once again their importance for increasing the hardness and cohesiveness from the production and human site (pet owner) perspective. The highly positive correlations found between initial crispiness with musty/dusty aroma and fracturability were mainly driven by the scores for the EP treatments. The panelists identified the EP treatments as hard and difficult to bite, with values of 13.0 and 14.5 out of 15.0 for the WWS and WRS, respectively. Peak bite forces in adult humans can go from 200 to 450 newtons (N) (Lieberman, 2011). As earlier stated, these treatments also presented eating difficulties for adult Beagle dogs. Adult dogs can have a wide range of bite forces. Lindner et al. (1995) evaluated 22 pet dogs between 7 and 55 kg and determined bite forces ranges from 13 to 1394 N with a mean of 256 N. The averaged value found in dogs closely resembled the values reported in humans; thus, the collective perception of the panelists served as an indicator of the force the dogs needed to exert in order to consume the treats.

The sensory relationships described by the panelists regarding various attributes for color, aroma, and hardness (brown appearance with musty/dusty aroma and initial crispiness, and toasted aroma with stale aroma) may have been associated with the Maillard reaction that occurred during baking and includes a group of reactions rather than a single reaction. In biscuit production, reducing sugars (monosaccharides and lactose) react with free amino acids (especially lysine) when the product is heated during baking and promotes the brown hue formation on the surface, contributing to the texture and flavor (Leiva-Valenzuela et al., 2018). It is important to emphasize that the Maillard reaction has also an effect on animal assimilation of the product as the bioavailability of lysine reduces (van Rooijen et al., 2013). Treats are products not intended to fulfill the nutritional requirements of the animal. Nonetheless, it would be recommended to quantify the reactive lysine to evaluate their nutritive value.

Similarly, the predominant “grainy” flavor detected and the strong positive correlations between the “grainy” aroma and the “overall intensity” aroma could be influenced by the formulation of the products in which the main ingredient was a cereal (wheat or sorghum). According to Ma et al. (2017), high-carbohydrate (human) food is usually related to sweet taste, while the savory taste is associated with high-protein food (Griffioen-Roose et al., 2012). Savory taste refers to nonsweet taste and it is closely linked to the “umami,” which is also described as a “broth-like” or “meaty” flavor (Yamaguchi and Ninomiya, 2000). In our evaluation, the sweet aromatics were only slightly perceived by the panelists, whereas the savory taste was not identified. Therefore, the soluble animal proteins in the amounts added did not overshadow the predominant “grainy” taste from the high level of cereals.

Commonly, sweet and umami tastes are well-accepted by dogs and humans because they are associated with nutritive foods (Houpt and Smith, 1981; Yarmolinsky et al., 2009). In addition, Houpt et al. (1979) described that female dogs tend to have a slightly more preference for sucrose as compared to males. Despite the differences reported among species in sweet taste receptors and genes that influence the sweet taste responses (Bachmanov et al., 2011), the scores obtained from the panelists gave us a narrower idea of the attributes which existed in these products. Nonetheless, further research should be conducted to better understand these observations.

### 4.3 Oxidation rate evaluation

Lipid oxidation is a process in which unsaturated fatty acids react with oxygen, creating intermediate products (lipid hydroperoxides) that are tasteless and odorless. These can be further decomposed into volatile compounds (aldehydes, ketones, and hydrocarbons) that interact with food components (Mozuraityte et al., 2016). The secondary volatile products are important quality indicators because they degrade food quality and influence the organoleptic, nutritional, and shelf-life properties of a product (Jeleń and Wąsowicz, 2011).

There are numerous methods to evaluate oxidation of fats. These range from measurement of compounds such as peroxide value which are an indicator of hydroperoxides, anisidine value which is an indicator of nonvolatile secondary oxidation products, and free fatty acids that are products of hydrolysis of the triglyceride and organic volatiles (Velasco et al., 2010; Mozuraityte et al., 2016; Bench, 2019 ). In raw and processed cereals, hexanal is often considered a good indicator of oxidation because of their high linoleic acid content (Gebreselassie and Clifford, 2016). Hexanal is a main product of n-6 polyunsaturated fatty acids oxidation with green and fat odor notes. In cooked products, it is mainly formed by auto-oxidation which occurs via a free-radical chain mechanism in an autocatalytic manner (Shahidi, 2001; Mozuraityte et al., 2016).

Oxidation can happen before and during the processing of biscuits. The oxidative stability of a product can be attributed to the formulation (moisture content, physical-chemical properties), the processing, the antioxidants included, the packaging (water, vapor, O_2_, or CO_2_ permeability), and storage conditions (temperature, light, humidity) (Galić et al., 2009). For this reason, some authors suggest analyzing the fat composition and level of oxidation that ingredients possess before making a product (Manzocco et al., 2020) because an ingredient with a very high oxidation level can lead to a rise in the level of primary oxidation products, and subsequently secondary oxidation products may accumulate after processing. Nonetheless, other authors have found that the ingredient oxidation does not completely account for later product deterioration (Gray, 2015).

It has been documented that there exists a high level of lipid oxidation in dough preparation due to the presence of active enzymes and oxygen available. It can also occur during baking, but in minor proportions (Caponio et al., 2008; Maire et al., 2013). Interestingly, the high baking temperatures can have a two-factor effect on a product. They can inactivate the enzymes responsible for oxidation (lipase and lipoxygenase) and also favor auto-oxidation (Maire et al., 2013). Additionally, the baking temperatures can produce Maillard Reactions Products (MRP) which to some degree are considered antioxidants (Barden and Decker, 2016). The MRP can act as oxygen scavengers or metal ion sequestrators, slowing the initial lipid oxidation and thereby hydroperoxide formation (Bressa et al., 1996).$

Wheat and sorghum contain low levels of total fats that vary from (2.2%–3.3%) and (3.9%), respectively. Additionally, the predominant fatty acids from wheat are linoleic (56.3%) and palmitic (24.5%); whereas, in sorghum, oleic and linoleic acids account for 84% of the total fatty acids making it highly unsaturated (Becker, 2007). In our study, the original level of hydroperoxides and secondary oxidation products was not analyzed in the ingredients before producing the treats. This could be why the initial hexanal level and stale (lack of freshness) aroma detected by the panelists were higher, especially in the EP treatments as the odor threshold for hexanal has been previously reported to be at 97 ppb in healthy adult (22–40 yr) humans (Ernstgård et al., 2017). At those quantified values, we also expected a canine perception because dogs’ olfactory receptors are more sensitive to hexanal than those from humans (Cho and Park, 2019); nonetheless, this note did not cause any refusal of the product but could instead influence the buying or serving decisions of the pet owner (Koppel et al., 2013).

Besides the WRS-GL and WRS-EP treatments that reduced hexanal content over time, most of the treatments had consistent values. This observation agreed with Mandić et al. (2013), who noted that hexanal content in refined and whole grain wheat and buckwheat crackers had values lower than 1.0 mg/kg until the sixth month. However, after that point, the values increased to > 5.0 mg/kg towards month 12 at ambient temperature (22 ± 2 °C). Similarly, Sakač et al. (2016) observed a similar pattern during the first 9 mo of unpacked and packed gluten-free rice-buckwheat cookies stored at 23 °C and 40% relative humidity for 16 mo. Nonetheless, these authors reported higher aldehydes values (2.05–3.93 mg/kg) when they combined the octanal, hexanal, and pentanal results. It is worth emphasizing that our study was conducted at a higher temperature and relative humidity and yet the treats had low hexanal values. Though the cited studies all evaluated products with higher fat content (>20%) and for more extended periods.

The reduction of hexanal observed in some treatments could be explained by the possibility that it volatilized through the holes in whirl bags or that some oxidative reactions involving hexanal occurred during the storage period. Similar findings were observed by Purcaro et al. (2008) when analyzed crispy bread for 12 mo at 39%–43% RH. However, further evaluation should be performed characterizing the spray-dried plasma, egg protein, and gelatin level on markers of oxidation, including other aldehydes such as heptanal, (t)-2-heptenal, nonanal, and (t)-2-nonenal.

Another factor that can influence oxidation is the level of iron in a product due to its ability to enhance the propagation of lipid peroxidation through redox cycling even at very low concentrations (<50 ppb). This reaction creates free radicals that further attack labile molecules (Minotti and Aust, 1992; Goddard et al., 2012). The manufacturers reported that the whole flours used in our study contained iron; thus, some oxidation was expected. However, our observations let us conclude that iron did not affect the oxidation stability of treats because the low moisture in the product most likely reduced its diffusion as reported by Barden (2014).

## 5. Conclusion$

Our hypothesis was validated through this study as the addition of soluble animal proteins enhanced the sensory attributes of sorghum rotary-molded dog treats. Moreover, the resultant treats were highly comparable to those made with wheat when SDP and GL were included. Results from the human sensory panel complemented the interpretation of the ranking test and better-defined differences in the product appearance and acceptability. There was not an impairment in the oxidation rates. The hexanal values were not affected when SDP or GL were included as compared to WWF-GTN (<1.0 mg/kg); however, the EP considerably increased the hexanal concentrations, especially at the beginning of the study and throughout the evaluation. It is recommended that another ranking test with a larger dog cohort and descriptive sensory analysis be performed over time to identify rancidity notes which would help predict shelf-life stability. Also, other aldehydes typical for rancidity development should be analyzed to identify the changes in their profile over a longer period.

## Abbreviations

AFT: aftertaste
APR: appearance
ARM: aroma
FVR: flavor
PCA: principal component analysis
TEX: texture

## References

[CIT0001] Aldrich, C. G., and K. Koppel. 2015. Pet food palatability evaluation: a review of standard assay techniques and interpretation of results with a primary focus on limitations. Anim. Open Access J. MDPI 5:43–55. doi:10.3390/ani5010043. PMC4494336 26479136

[CIT0002] Alegría-Morán, R. A., S. A. Guzmán-Pino, J. I. Egaña, C. Muñoz, and C. J. Figueroa. 2019. Food preferences in dogs: effect of dietary composition and intrinsic variables on diet selection. Anim. Open Access J. MDPI 9:1–12. doi:10.3390/ani9050219. PMC6562821 31064159

[CIT0003] Araujo, J. A., and N. W. Milgram. 2004. A novel cognitive palatability assessment protocol for dogs. J. Anim. Sci. 82:2200–2206. doi:10.2527/2004.8272200x. 15309970

[CIT0004] Awika, J. M., and L. W. Rooney. 2004. Sorghum phytochemicals and their potential impact on human health. Phytochem. 65:1199–1221. doi:10.1016/j.phytochem.2004.04.001. 15184005

[CIT0005] Bachmanov, A. A., N. P. Bosak, W. B. Floriano, M. Inoue, X. Li, C. Lin, V. O. Murovets, D. R. Reed, V. A. Zolotarev, and G. K. Beauchamp. 2011. Genetics of sweet taste preferences. Flavour Fragr. J. 26:286–294. doi:10.1002/ffj.2074. 21743773 PMC3130742

[CIT0006] Barden, L. M. 2014. Understanding lipid oxidation in low-moisture food. University of Massachusetts Amherst. doi:10.7275/RK58-7Z49

[CIT0007] Barden, L., and E. A. Decker. 2016. Lipid oxidation in low-moisture food: a review. Crit. Rev. Food Sci. Nutr. 56:2467–2482. doi:10.1080/10408398.2013.848833. 24279497

[CIT0008] Becker, R. 2007. Fatty acids in food cereal grains and grain products. In C. Kuang Chow, editor, Fatty acids in foods and their health implications. 3rd ed. Vol. 20073230. CRC Press. p. 303–316. doi:10.1201/9781420006902.ch12

[CIT0009] Bench, B. J. 2019. Determination of lipid oxidation parameters in solid non-oil matrices and the impacts on the pet food industry. AOAC Annual Meeting & Expo. p. 1.

[CIT0010] Bhadra, A., and A. Bhadra. 2014. Preference for meat is not innate in dogs. J. Ethol. 32:15–22. doi:10.1007/s10164-013-0388-7.

[CIT0011] Bradshaw, J. W. 2006. The evolutionary basis for the feeding behavior of domestic dogs (Canis familiaris) and cats (Felis catus). J. Nutr. 136:1927S–1931S. doi:10.1093/jn/136.7.1927S. 16772461

[CIT0012] Bramoulle, L., J. Ruaud, I. Guiller, and A. Levesque. 2013. Method for producing highly palatable dry cat food (Justia Patents Patent No. 20130287930).https://patents.justia.com/patent/20130287930

[CIT0013] Bressa, F., N. Tesson, M. Dalla Rosa, A. Sensidoni, and F. Tubaro. 1996. Antioxidant effect of maillard reaction products: application to a butter cookie of a competition kinetics analysis. J. Agric. Food Chem. 44:692–695. doi:10.1021/jf950436b.

[CIT0014] Caponio, F., C. Summo, A. Pasqualone, and M. T. Bilancia. 2008. Effect of kneading and baking on the degradation of the lipid fraction of biscuits. J. Cereal Sci. 48:407–412. doi:10.1016/j.jcs.2007.11.003.

[CIT0015] Case, L., L. Daristole, M. Hayek, and M. Raasch. 2011. Digestion and absorption. In: Canine and feline nutrition. 3rd ed. Elsevier. p. 45–53.

[CIT0016] Castillo, M. 2014. The complicated equation of smell, flavor, and taste. . AJNR Am. J. Neuroradiol. 35:1243–1245. doi:10.3174/ajnr.A3739. 24091442 PMC7966593

[CIT0017] Chiremba, C., R. N. Taylor, and K. G. Duodu. 2009. Phenolic content, antioxidant activity, and consumer acceptability of sorghum cookies. Cereal Chem. 86:590–594. doi:10.1094/CCHEM-86-5-0590.

[CIT0018] Cho, S. W., and T. H. Park. 2019. Comparative evaluation of sensitivity to hexanal between human and canine olfactory receptors. Biotechnol. Bioprocess Eng. 24:1007–1012. doi:10.1007/s12257-019-0265-5.

[CIT0019] Di Donfrancesco, B., K. Koppel, and E. Chambers. 2012. An initial lexicon for sensory properties of dry dog food. J. Sens. Stud. 27:498–510. doi:10.1111/joss.12017.

[CIT0020] Di Donfrancesco, B., K. Koppel, M. Swaney-Stueve, and E. Chambers. 2014. Consumer acceptance of dry dog food variations. Anim. Open Access J. MDPI 4:313–330. doi:10.3390/ani4020313. PMC4494379 26480043

[CIT0021] Dornblaser, L. 2017. What human food trends mean for treats and toppers. https://www.k-state.edu/pet-food/events/docs/2017/Dornblaser.pdf.

[CIT0022] Ernstgård, L., A. M. Dwivedi, J. N. Lundström, and G. Johanson. 2017. Measures of odor and lateralization thresholds of acrolein, crotonaldehyde, and hexanal using a novel vapor delivery technique. PLoS One 12:e0185479. 1–14. doi:10.1371/journal.pone.0185479. 28950007 PMC5614536

[CIT0023] Francis, J., K. Thompson-Witrick, and E. B. Perry. 2020. 104 president oral presentation pick: sensory analysis of horse treats: a comparison between horses and humans. J. Anim. Sci. 98 (Suppl. 4):91. (Abstr.). doi:10.1093/jas/skaa278.166.

[CIT0024] Galić, K., D. Ćurić, and D. Gabrić. 2009. Shelf life of packaged bakery goods—a review. Crit. Rev. Food Sci. Nutr. 49:405–426. doi:10.1080/10408390802067878. 19399669

[CIT0025] Gebreselassie, E., and H. Clifford. 2016. Oxidative stability and shelf life of crackers, cookies, and biscuits. In: Oxidative stability and shelf life of foods containing oils and fats. Elsevier. p. 461–478. doi:10.1016/B978-1-63067-056-6.00012-4.

[CIT0026] Goddard, J. M., D. J. McClements, E. A. Decker. 2012. Innovative technologies in the control of lipid oxidation. Lipid Technol. 24:275–277. doi:10.1002/lite.201200242.

[CIT0027] Gray, M. 2015. Evaluation of oxidized rendered protein meals in pet foods. Kansas State University.

[CIT0028] Griffin, R. W., and L. M. Beidler. 1984. Studies in canine olfaction, taste and feeding: A summing up and some comments on the academic-industrial relationship. Neurosci. Biobehav. Rev. 8:261–263. doi:10.1016/0149-7634(84)90050-2. 6462557

[CIT0029] Griffioen-Roose, S., M. Mars, E. Siebelink, G. Finlayson, D. Tomé C. de Graaf. 2012. Protein status elicits compensatory changes in food intake and food preferences123. Am. J. Clin. Nutr. 95:32–38. doi:10.3945/ajcn.111.020503. 22158729 PMC3238463

[CIT0030] Hall, N., F. Peron, S. Cambou, L. Callejón, and C. Wynne. 2017. Food and food-odor preferences in dogs: a pilot study. Chem. Senses 42:361–370. doi:10.1093/chemse/bjx016.

[CIT0031] Hall, J. A., J. C. Vondran, M. A. Vanchina, D. E. Jewell. 2018. When fed foods with similar palatability, healthy adult dogs and cats choose different macronutrient compositions. J. Exp. Biol. 221:jeb173450. 1–14. doi:10.1242/jeb.173450. 29773684

[CIT0032] Hidalgo, M., and C. Takatsu. 2012. Desarollo de un snack para canes. Universidad San Francisco de Quito. http://repositorio.usfq.edu.ec/bitstream/23000/1918/1/103475.pdf

[CIT0033] Houpt, K. A., B. Coren, H. F. Hintz, J. E. Hilderbrant. 1979. Effect of sex and reproductive status on sucrose preference, food intake, and body weight of dogs. J. Am. Vet. Med. Assoc. 174:1083–1085. 571424

[CIT0034] Houpt, K., P. Davis, and H. Hintz. 1982. Effect of peripheral anosmia in dogs trained as flavor validators. Am. J. Vet. Res. 43:841–843. 7091848

[CIT0035] Houpt, K. A., and S. L. Smith. 1981. Taste preferences and their relation to obesity in dogs and cats. Can. Vet. J. 22:77–81. 7248879 PMC1789883

[CIT0036] House, L. R., M. Osmanzai, M. I. Gomez, and E. S. Monyo. 1995. Agronomic principles. In: D.A. Dendy, editor, Sorghum and millets. Chemistry and technology. American Association of Cereal Chemists, Inc. St. Paul, MN. p. 27–67.

[CIT0037] Jeleń, H., and E. Wąsowicz. 2011. Lipid-derived flavor compounds. In: H. Jelen, editor, Food flavors. Vol. 20116950. CRC Press. p. 65–94. doi:10.1201/b11187-5.

[CIT0038] Kim, S. E., B. Arzi, T. C. Garcia, F. J. Verstraete. 2018. Bite forces and their measurement in dogs and cats. Front. Vet. Sci. 5: 1–6. doi:10.3389/fvets.2018.00076. 29755988 PMC5932386

[CIT0039] Kitchell, R. L. 1972. Dogs know what they like. Friskies Res. 8:1–4.

[CIT0040] Koppel, K. 2014. Sensory analysis of pet foods. J. Sci. Food Agric. 94:2148–2153. doi:10.1002/jsfa.6597. 24497160

[CIT0041] Koppel, K., K. Adhikari, and B. Di Donfrancesco. 2013. Volatile compounds in dry dog foods and their influence on sensory aromatic profile. Molecules 18:2646–2662. doi:10.3390/molecules18032646. 23446921 PMC6270422

[CIT0042] Leiva-Valenzuela, G. A., M. Quilaqueo, D. Lagos, D. Estay, F. Pedreschi. 2018. Effect of formulation and baking conditions on the structure and development of non-enzymatic browning in biscuit models using images. J. Food Sci. Technol. 55:1234–1243. doi:10.1007/s13197-017-3008-7. 29606738 PMC5876191

[CIT0043] Lema, K. A. 2021. Supplementation of gluten-free sorghum flour-based dog treats with soluble animal proteins. Kansas State University. https://krex.k-state.edu/dspace/bitstream/handle/2097/41281/KrystinaLemaAlmeida2021.pdf?sequence=1&isAllowed=y

[CIT0044] Li, H., S. Smith, C. G. Aldrich K. Koppel. 2017. Preference ranking procedure proposal for dogs: a preliminary study. J. Sens. Stud. 33:e12307. 1–10. doi:10.1111/joss.12307.

[CIT0045] Lieberman, D. E. 2011. The evolution of the human head. Harvard University Press.

[CIT0046] Lindner, D. L., S. M. Marretta, G. J. Pijanowski, A. L. Johnson C. W. Smith. 1995. Measurement of bite force in dogs: a pilot study. J. Vet. Dent. 12:49–52. doi:10.1177/089875649501200202. 9693626

[CIT0047] Ma, Y., R. Ratnasabapathy, and J. Gardiner. 2017. Carbohydrate craving- not everything is sweet. Curr. Opin Clin. Nutr. Metab. Care 20:261–265. doi:10.1097/MCO.0000000000000374. 28375878 PMC5837018

[CIT0048] Maire, M., B. Rega, M. E. Cuvelier, P. Soto P. Giampaoli. 2013. Lipid oxidation in baked products: impact of formula and process on the generation of volatile compounds. Food Chem. 141:3510–3518. doi:10.1016/j.foodchem.2013.06.039. 23993514

[CIT0049] Mandić, A. I., I. J. Sedej, M. B. Sakač, and A. C. Mišan. 2013. Static headspace gas chromatographic method for aldehyde determination in crackers. Food Anal. Methods 6:61–68. doi:10.1007/s12161-012-9415-5.

[CIT0050] Manzocco, L., G. Romano, S. Calligaris, and M. C. Nicoli. 2020. Modeling the effect of the oxidation status of the ingredient oil on stability and shelf life of low-moisture bakery products: the case study of crackers. Foods 9: 1–13. doi:10.3390/foods9060749. PMC7353518 32517073

[CIT0051] Minotti, G., and S. D. Aust. 1992. Redox cycling of iron and lipid peroxidation. Lipids 27:219–226. doi:10.1007/BF02536182. 1326072

[CIT0052] Mozuraityte, R., V. Kristinova, and T. Rustad. 2016. Oxidation of food components. In Encyclopedia of food and health. Elsevier. p. 186–190. doi:10.1016/B978-0-12-384947-2.00508-0.

[CIT0053] Nagodawithana, T. W., L. Nelles , and N. B. Trivedi. 2008. Protein hydrolysates as hypoallergenic, flavors and palatants for companion animals. In: V. K. Pasupuleti and A. L. Demain, editors, Protein hydrolysates in biotechnology. Springer Netherlands. p. 191–207. doi:10.1007/978-1-4020-6674-0_11.

[CIT0054] Pétel, C., C. Baron, M. Thomsen, L. Callejon, and F. Péron. 2018. A new method to assess the influence of odor on food selection in dogs. J. Sens. Stud. 33:e12311. 1–7. doi:10.1111/joss.12311.

[CIT0055] Pétel, C., P. Pachot, L. Bramoullé, S. Cambou , and L. Callejón. 2018. Moisture impact on kibble parameters and dog’s sensory perception [Poster]. EFFOST. https://content.diana-petfood.com/plezi_file/5cfa2a4654067a5202085db3/diana-pet-food-scientific-poster-dog-kibble-moisture.pdf

[CIT0056] Purcaro, G., S. Moret, and L. S. Conte. 2008. HS–SPME–GC applied to rancidity assessment in bakery foods. Eur. Food Res. Technol. 227:1–6. doi:10.1007/s00217-007-0715-8.

[CIT0057] Rashotte, M. E., and J. C. Smith. 1984. Operant conditioning methodology in the assessment of food preferences: introductory comments. Neurosci. Biobehav. Rev. 8:211–215. doi:10.1016/0149-7634(84)90043-5. 6462550

[CIT0058] Sakač, M., M. Pestorić, A. Mandić, A. Mišan, N. Nedeljković, D. Jambrec, P. Jovanov, V. Lazić, L. Pezo, et al. 2016. Shelf-life prediction of gluten-free rice-buckwheat cookies. J. Cereal Sci. 69:336–343. doi:10.1016/j.jcs.2016.04.008.

[CIT0059] Shahidi, F. 2001. Headspace volatile aldehydes as indicators of lipid oxidation in foods. In: R. L. Rouseff and K. R. Cadwallader, editors, Headspace analysis of foods and flavors. Vol. 488. Springer US. p. 113–123. doi:10.1007/978-1-4615-1247-9_9. 11548150

[CIT0060] Sprinkle, D. 2019. Current top trends in pet treats and chews. https://www.petfoodindustry.com/articles/8573-current-top-trends-in-pet-treats-and-chews

[CIT0061] Surie, M. L. 2014. An exploratory study on the pet food purchasing behaviour of New Zealand consumers. Lincoln University. https://core.ac.uk/download/pdf/35469503.pdf

[CIT0062] Thompson, H., S. Riemer, S. L. Ellis, O. H. Burman. 2016. Behaviour directed towards inaccessible food predicts consumption—a novel way of assessing food preference. Appl. Anim. Behav. Sci. 178:111–117. doi:10.1016/j.applanim.2016.02.008.

[CIT0063] Tobie, C., F. Péron, and C. Larose. 2015. Assessing food preferences in dogs and cats: a review of the current methods. Anim. Open Access J. MDPI 5:126–137. doi:10.3390/ani5010126. PMC4494339 26479142

[CIT0064] Turek, J. J., B. A. Watkins, I. A. Schoenlein, K. G. Allen, M. G. Hayek C. G. Aldrich. 2003. Oxidized lipid depresses canine growth, immune function, and bone formation. J. Nutr. Biochem. 14:24–31. doi:10.1016/S0955-2863(02)00221-8. 12559474

[CIT0065] van Rooijen, C., G. Bosch, A. F. Poel, P. A. van der, Wierenga, L. Alexander W. H. Hendriks. 2013. The Maillard reaction and pet food processing: effects on nutritive value and pet health. Nutr. Res. Rev. 26:130–148. doi:10.1017/S0954422413000103. 23916186

[CIT0066] Velasco, J., C. Dobarganes, and G. Márquez-Ruiz. 2010. Oxidative rancidity in foods and food quality. In: Chemical deterioration and physical instability of food and beverages. Elsevier. p. 3–32. doi:10.1533/9781845699260.1.3.

[CIT0067] Wang, K. J., Y. H. Lee, and F. Kurniawan. 2012. Evaluation criteria of new product development process—a comparison study between Indonesia and Taiwan industrial manufacturing firms. Int. J. Innov. Manag. 16:1250021. 1–27. doi:10.1142/S1363919612003824.

[CIT0068] Yamaguchi, S., and K. Ninomiya. 2000. Umami and food palatability. J. Nutr. 130:921S–926S. doi:10.1093/jn/130.4.921S. 10736353

[CIT0069] Yarmolinsky, D. A., C. S. Zuker N. J. Ryba. 2009. Common sense about taste: from mammals to insects. Cell 139:234–244. doi:10.1016/j.cell.2009.10.001. 19837029 PMC3936514

[CIT0070] Young, N. W. 2011. The influence of ingredients on product stability and shelf life. In: Food and beverage stability and shelf life. Woodhead Publishing Series in Food Science, Technology and Nutrition. p. 132–183. doi:10.1533/9780857092540.1.132.

[CIT0071] Zoon, H., C. de Graaf, and S. Boesveldt. 2016. Food odours direct specific appetite. Foods 5:12. 1–10. doi:10.3390/foods5010012. PMC5224573 28231107

